# Fisetin: An anticancer perspective

**DOI:** 10.1002/fsn3.1872

**Published:** 2020-11-25

**Authors:** Muhammad Imran, Farhan Saeed, Syed Amir Gilani, Mohammad Ali Shariati, Ali Imran, Muhammad Afzaal, Muhammad Atif, Tabussam Tufail, Faqir M. Anjum

**Affiliations:** ^1^ Faculty of Allied Health Sciences University Institute of Diet and Nutritional Sciences The University of Lahore Lahore Pakistan; ^2^ Institute of Home & Food Sciences Government College University Faisalabad Pakistan; ^3^ Laboratory of Biocontrol and Antimicrobial Resistance Orel State University Named After I.S. Turgenev Orel Russia; ^4^ Department of Clinical Laboratory Sciences College of Applied Medical Sciences Jouf University Sakaka Saudi Arabia; ^5^ The University of Gambia Sere Kunda Gambia

**Keywords:** anticancer, breast cancer, chemopreventive agent, fisetin, flavonoids

## Abstract

Despite the provision of safe and cost‐effective chemopreventive cancer approaches, still there are requirements to enhance their efficiency. The use of dietary agents as phytochemicals plays an imperative role against different human cancer cell lines. Among these novel dietary agents, fisetin (3,3′,4′,7‐tetrahydroxyflavone) is present in different fruits and vegetables such as apple, persimmon, grape, strawberry, cucumber, and onion. Being a potent anticancer agent, fisetin has been used to inhibit stages in the cancer cells (proliferation, invasion), prevent cell cycle progression, inhibit cell growth, induce apoptosis, cause polymerase (PARP) cleavage, and modulate the expressions of Bcl‐2 family proteins in different cancer cell lines (HT‐29, U266, MDA‐MB‐231, BT549, and PC‐3M‐luc‐6), respectively. Further, fisetin also suppresses the activation of the PKCα/ROS/ERK1/2 and p38 MAPK signaling pathways, reduces the NF‐κB activation, and down‐regulates the level of the oncoprotein securin. Fisetin also inhibited cell division and proliferation and invasion as well as lowered the TET1 expression levels. The current review article highlights and discusses the anticancer role of fisetin in cell cultures and animal and human studies. Conclusively, fisetin as a polyphenol with pleiotropic pharmacological properties showed promising anticancer activity in a wide range of cancers. Fisetin suppresses the cancer cell stages, prevents progression in cell cycle and cell growth, and induces apoptosis.

## Graphical abstract

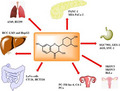

## 1 INTRODUCTION

Flavonoids are secondary metabolites that belong to polyphenolic class, mostly present in different fruits and vegetables. Due to low toxicity, flavonoid compounds have been known to play a significant role in the biological systems and exhibited wide range of health‐endorsing perspectives in various in vitro and in vivo studies (Abubakar, Abdullah, Sulaiman, & Suen, 2012; Benavente‐Garcia & Castillo, 2008; Chaudhuri, Banerjee, Basu, Sengupta, & Sengupta, 2007). Fisetin is an important phytoflavonoid that possesses very good antioxidant, anti‐Parkinson's, and anticancer activities. Multiple studies of different researchers investigated and expounded the chemopreventive role of flavonoid moieties against various types of human cancers (Kumar et al., 2020). Among flavonoids, fisetin (3,7,3′,4′‐tetrahydroxyflavone 1) is generally recognized as a bioactive plant, which has been used as potential drug against various free radical mediated as well as human cancers (Sengupta, Banerjee, & Sengupta, 2005). Fisetin is frequently present in vegetables (onions and cucumbers), fruits (persimmon, apples, strawberries), wine, and nuts, and their concentrations are varied from 2 to 160 mg/g with an average daily intake estimate of 0.4 mg. Nutritional supplements are also prepared by fisetin by using higher concentrations and exhibited different pharmacological activities, including antioxidant and anti‐inflammatory activity acting. Due to scavenging and neutralizing effects of fisetin, it also induces apoptosis, exhibits cell cycle hold, and suppresses the cyclin‐dependent kinases (CDKs) in human cancer cell lines. Fisetin also has been known to modulate the lipid kinase and protein kinase pathways (Inkielewicz‐Stepniak, Radomski, & Wozniak, 2012; Liao, Shih, Chao, Lee, & Chiang, 2009; Park et al., 2008).

In human multiple myeloma U266 cells, fisetin stimulated the production of free radical species that led to apoptosis signaling as well as showed AMP‐activated protein kinase signaling (Jang et al., 2012). Multiple studies also authenticated the anticancer role of fisetin through various signaling pathways such as blocking of mammalian target of rapamycin (PI3K/Akt/mTOR)/phosphatidylinositol‐3‐kinase/protein kinase B, mitogen‐activated protein kinases (MAPK)‐dependent nuclear factor kappa‐light‐chain‐enhancer of activated B cells (NF‐κB), and p38, respectively, whereas these cellular processes have significantly led to malignancy (Mukhtar, Adhami, Sechi, & Mukhtar, 2015; Ren et al., 2020).

## 2 CANCER PERSPECTIVES

### 2.1 Breast cancer

Epigenetic regulation of human epidermal growth factor receptor 2 (HER2) is observed in breast cancer. Fisetin has shown the effects on human epidermal growth factor receptor 2 (HER2)/neu‐overexpressing breast cancer cell lines. Fisetin caused induction through inactivating the receptor, inducing the degradation of the proteasomes, reducing its half‐life, reducing phosphorylation enolase, and altering the phosphatidylinositol 3‐kinase/Akt signaling (Guo, Dong, & Shi, 2018). In triple‐negative breast cancer (TNBC) cell lines, MDA‐MB‐231 and BT549 cells, fisetin in dose‐dependent concentration has been found to suppress the cell proliferation, migration, and invasion. In addition, mutation of epithelial‐to‐mesenchymal transition (EMT), inhibition of phosphoinositide 3‐kinase (PI3K)‐Akt‐GSK‐3β signaling pathway, and up‐regulation of expression of PTEN mRNA and protein were reported after fisetin treatment (Li et al., 2018).

In breast cancer cells (4T1 and JC cells), fisetin increased HO‐1 mRNA and protein expressions, elevated Nrf2 expression, and abrogated the HO‐1 expression, whereas HO‐1 expression was mediated by up‐regulation of the transcription factor Nrf2. In addition, fisetin reduced MMP‐2 and MMP‐9 enzyme activity and gene expression for both mRNA levels and protein (Tsai et al., 2018).

Different mechanisms are linked with preventive role of fisetin against the proliferation of breast cancer cell lines such as MDA‐MB‐231, MCF‐7, and 4T1. Fisetin, a natural flavonoid, was found to prevent the various stages of cancer (migration and invasion). In in vitro study of mice, the effects of fisetin led to prohibition of metastasis and invasiveness, induction of the apoptosis, and regulation of the phosphatidylinositol‐3‐kinase/protein kinase B/mammalian target of rapamycin pathway, whereas suppression of growth of breast tumors and enhancement of tumor cell apoptosis were observed in serum of tumor‐bearing mice (Sun et al., 2018).

Many recent reports elaborated the potential and significant role of fisetin against human breast cancer MCF‐7 cell proliferation, and they found that fisetin decreased the growth and development and also induced apoptotic cell death (Pawar, Singh, Rajalakshmi, Shaikh, & Bothiraja, 2018). Fisetin evidently suppressed the tumor burden, migration, and volume in MCF‐7 breast cancer cells as observed by Wang, Zhang, and Wang (2017). The mechanism of growth inhibition of MDA‐MB‐468 and MDA‐MB‐231 TNBC cells by fisetin treatment was explored; it was shown that fisetin prevented the activity of the estrogen receptor (MCF‐7 cells), colony formation, human epidermal growth factor receptor 2‐overexpressing (SK‐BR‐3 cells), cell division (TNBC cells), and induced apoptosis. In addition, fisetin treatment further led to permeabilization of mitochondrial membrane, activation of caspase‐8 and caspase‐9, as well as the cleavage of poly(ADP‐ribose) polymerase 1. Moreover, it also induced caspase‐dependent apoptosis, lowered phosphorylation of histone H3 at serine 10, caused G2/M phase of the cell cycle, and suppressed the Aurora B kinase, respectively, in above‐mentioned human cancer cell lines (Smith, Murphy, Doucette, Greenshields, & Hoskin, 2016). Our data showed that significant attenuation of 12‐O‐tetradecanoylphorbol‐13‐acetate (TPA)‐induced cell invasion, suppression of activation of the PKCα/ROS/ERK1/2 and p38 MAPK signaling pathways, and reduction in NF‐κB activation were reported after fisetin treatment. However, these changes led to the down‐regulation of matrix metalloproteinase (MMP)‐9 expression in MCF‐7 human breast cancer cells (Noh et al., 2015). In human breast cancer MCF‐7, we validated the preventive role of fisetin against cancer proliferation in MDA‐MB‐231 cells through previous findings. They examined that fisetin showed numerous apoptotic characteristics such as breakup of plasma membrane, mitochondrial depolarization, caspase (−7, −8, and −9) activation and PARP cleavage, p53 activation, and autophagy reduction. In contrast, fisetin also performed neither other features nor such fragmentation of DNA and phosphatidylserine (PS) externalization (Yang, Tseng, Peng, Chen, & Chiu, 2012).

### 2.2 Prostate cancer

The combining effect of fisetin (20μmol/L) and cabazitaxel (5 nmol/L) significantly decreased cell viability and metastatic properties of different cancer cell lines such as PC‐3M‐luc‐6, 22Rν1, and C4‐2 on normal prostate epithelial cells. Additionally, both compounds inhibited the cancer stages (proliferation, tumor growth, and invasion) and induced apoptosis in in vivo xenograft mouse models (Mukhtar, Adhami, Siddiqui, Verma, & Mukhtar, 2016). Fisetin treatment resulted in prostate cancer (PCa) of in vitro and in vivo xenograft animals caused down‐regulation of intracellular and secreted hyaluronan levels and suppressed the hyaluronan (HA) synthesis and degradation enzymes (Lali et al., 2016). Multiple pathways are involved to prevented from the proliferation of human prostate cancer cell lines through fisetin administration. The changes occurred are inhibition of viability and colony formation, robust up‐regulation of microtubule‐associated proteins (MAP)‐2 and MAP‐4, suppression of the migration and invasion stages, and inhibition of protein Nudc, which is linked with microtubule motor dynein complex that regulates microtubule dynamics (Mukhtar et al., 2015).

Epithelial‐to‐mesenchymal transition (EMT) and transcription/translation regulatory Y‐box binding protein‐1 (YB‐1) have imperative role in association with cancer metastasis. In human prostate cancer tissues, enhancement of YB‐1 expression was linked with tumor grade, while it exhibited an inverse relationship with E‐cadherin. The induction of mesenchymal morphology via forced YB‐1 expression was linked with epithelial markers that are controlled down. In PCa cells, ridiculing of YB‐1 reversed mesenchymal characteristics and reduced cell proliferation, migration, and invasion. Within the cold shock domain (CSD), YB‐1 is stimulated directly through Akt‐mediated phosphorylation at Ser102. Computational docking and molecular dynamics indicated that fisetin binds on CSD residues from β1 to β4 strands, inhibiting the interaction between Akt and YB‐1. Calculated free binding energy ranged from −11.9845 to −9.6273 kcal/mol. Fisetin binds to YB‐1 with an average affinity of 35 μM, in both slow association and dissociation. Fisetin also prevented EGF‐induced YB‐1 phosphorylation and EMT markers both in vitro and in vivo (Adhami, Syed, Khan, & Mukhtar, 2012; Khan, Afaq, Syed, & Mukhtar, 2008). A study conducted by Szliszka, Helewski, Mizgala, and Krol (2011) investigated that fisetin treatment in prostate cancer cell lines (LNCaP, DU145, and PC3) sensitized the tumor necrosis factor‐related apoptosis‐inducing ligand (TRAIL)‐resistant HepG2 cell. The augmentation of TRAIL‐mediated cytotoxicity and apoptosis through engaging the extrinsic (receptor‐mediated) and intrinsic (mitochondrial) apoptotic pathways was observed after fisetin treatment. Furthermore, it also lowered the activity of NF‐κB, caused significant activation of caspase‐8 and caspase‐3 and disruption of ΔΨm, and enhanced the levels of TRAIL‐R1, respectively (Szliszka et al., 2011). A study performed by Haddad et al., 2010 explored that fisetin dose‐dependently in PC3 and LNCaP cells at the rate of 1–50 microM significantly decreased the cell viability along with enhancement in apoptotic cell death as well as alterations of gene expressions. Moreover, the alteration in chromosome organization, apoptosis, and stress response was also observed (Haddad et al., 2010).

The targeting of mammalian target of rapamycin (mTOR) kinase and PTEN/PI3K/Akt are an important strategy to prevented from the proliferation of prostate cancer. Being a chemopreventive role against different cancer cell lines, fisetin has been found to suppress the PTEN/PI3K/Akt and mTOR signaling pathway. The down‐regulation of PRAS40, Rictor, Raptor, and GbetaL, which further led to loss of mTOR complex (mTORC)1/2 formation, activation of mTOR repressor TSC2 by suppressing Akt and activating AMPK, suppression of Cap‐dependent translation, and hypophosphorylation of 4EBP1 as well as induction of autophagic‐programmed cell death, was reported after fisetin treatment (Suh et al., 2010). A peer group of researchers and investigators explored that multiple processes are involved to prevented from the proliferation of prostate cancer such as suppression of MMP‐2 and MMP‐9 activities, inhibition of phosphorylation of c‐Jun N‐terminal kinases 1 and 2 (JNK1/2) and Akt, and reduction in c‐Fos, NF‐kB, and c‐Jun, as well as showed binding abilities of NF‐kappaB and activator protein‐1 (AP‐1), respectively (Chien, Shen, Huang, Ko, & Shih, 2010). Therefore, data reported from many studies about anticancer role of fisetin against different prostate cancer cell lines (CWR22Rupsilon1, LNCaP, and PC‐3) investigated that treatment of fisetin with different doses (10–60 microM, 48 hr) significantly found to lower the viability and exhibit cell cycle arrest at G(1) phase, momentous reduction in levels of protein (cyclins D1, D2, and E), and their activating partner cyclin‐dependent kinases 2, 4, and 6 with concomitant induction of WAF1/p21 and KIP1/p27. Moreover, it also induced apoptotic cell death and poly(ADP‐ribose) polymerase (PARP) cleavage, suppressed the phosphatidylinositol 3‐kinase and phosphorylation of Akt at Ser (473) and Thr (308), and modulated the levels of Bcl‐2 family proteins. Moreover, multiple pathways are also participated such as up‐regulation of second mitochondria‐derived activator of caspase, mitochondrial releasing of cytochrome c into cytosol, up‐regulation and down‐regulation of X‐linked inhibitor of apoptosis protein, and activation of caspase‐3, caspase‐8, and caspase‐9, accordingly (Khan et al., 2008).

### 2.3 Pancreatic cancer

**FIGURE 1:**
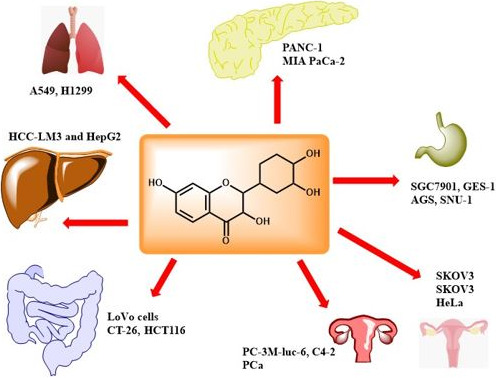
Mechanism of fisetin against cancer cells

Fisetin in murine xenograft pancreatic cancer PANC‐1 cells inhibits PANC‐1 cell proliferation, enhances AMPK/mTOR signaling pathway and stress‐induced transcription factor p8, induces autophagy (Figure 1), and increases the ATF4 and ATF6 (Jia et al., 2019).

Fisetin in combination with gemcitabine has performed potential anticancer role against pancreatic cancer cell lines (MIA PaCa‐2) through multiple approaches including suppression of tumor growth and proliferation, induction of apoptotic cell death, activation of caspase‐3/caspase‐7, reduction in ERK‐induced MYC instability at the protein level, down‐regulation of ERK at the protein and mRNA levels, and inhibition of extracellular signal‐regulated kinase (ERK)‐v‐myc avian myelocytomatosis viral oncogene homolog (MYC) pathway signaling, respectively (Kim et al., 2018; Youns & Abdel Halim Hegazv, 2018). Data reported by Murtaza and their colleagues investigated that fisetin treatment dose‐dependently in AsPC‐1 cells showed multiple anticancer mechanisms such as inhibition of cell growth, proliferation of cells, induction of apoptosis and invasion, suppression of DR3‐mediated NF‐kappaB activation, modulation of expressions of genes at the transcriptional level, enhancement of expression levels of IkappaBalpha, and down‐regulation of DR3 accompanied by activated pIkBalpha/beta kinases (pIKK's), pNF‐kappaB/p65, MMP9, and XIAP, respectively. In addition, fisetin markedly caused reduction in NF‐kappaB and pNF‐kappaB deoxyribonucleic acid (DNA)‐binding activity, NF‐kappaB promoter activity, and MMP9 promoter activity, respectively (Murtaza, Adhami, Hafeez, Saleem, & Mukhtar, 2009).

### 2.4 Colon

In in vitro and in vivo studies, fisetin works as an effective anticancer agent via inhibiting cell viability and tumor growth, inducing apoptosis, promoting caspase‐8 and cytochrome c expressions, and suppressing the aberrant activation of IGF1R and AKT proteins in LoVo cells of athymic nude mouse xenograft model (Jeng et al., 2018). Various in vivo studies showed that fisetin alone performed anticancer activities against human colon cancer cell lines (CT‐26 and HCT116) and markedly down‐regulated the level of the oncoprotein securin in a p53‐independent manner, whereas fisetin in combination with 2‐Gy radiation did not significantly suppress securin‐null HCT116 tumor growth compared with normal HCT116 tumors (Leu et al., 2016). Similarly, a study investigated after treatment of fisetin against CT26 cells concluded that fisetin exhibited multiple processes including suppression of tumor growth and proliferation, and enhancement of survival time, apoptotic cell death, and antiangiogenesis activities, respectively (Chen, Wu, et al., 2015; Wu, Lien, Shen, Yang, & Chen, 2014). Researchers also found that fisetin in combination with geldanamycin or radicicol in vivo study in human COLO205 colon cancer cells significantly increased expressions of caspase‐3 and caspase‐9 activities, modified the Bcl‐2 but not Bax protein or Bcl‐XL, and decreased the p53 protein along with enhancement in ubiquitin‐tagged proteins, respectively (Wu, Lien, Shen, Yang, & Chen, 2013; Yu, Yang, Peng, Yu, & Chiu, 2011). In HT‐29 colon cancer cells, supplementation of fisetin at the rate of 20–60 micromol/l caused cell cycle capture, inhibited the cyclin‐dependent kinase activities, and induced cleavage of poly(ADP‐ribose) polymerase (PARP), DNA condensations, and cleavage of caspase‐9, caspase‐7, and caspase‐3. These different doses of fisetin markedly increased the pro‐apoptotic levels Bak and Bim and reduced the protein levels of antiapoptotic Bcl‐xL and Bcl‐2. In addition, fisetin induced the mitochondrial translocation of Bax protein levels and release of cytochrome c and Smac/Diablo, and increased the permeability of the mitochondrial membrane. In addition, an enhancement in protein levels of cleaved caspase‐8, death receptor 5, Fas ligand, and TNF‐related apoptosis‐inducing ligand were reported after fisetin treatment, while caspase‐8 inhibitor Z‐IETD‐FMK inhibited the activation of caspase‐3 and fisetin‐induced apoptosis. Moreover, fisetin also enhanced the protein p53 levels and suppression expressions of p53 (Lim & Park, 2009). Suh and their coworkers investigated the anticancer potential of fisetin compound by applying different concentrations (30–120 microM) against HT29 colon cancer cells. The induction of apoptosis, suppression of the secretion of prostaglandin E2, down‐regulation of COX2 protein expression, and inhibition of Wnt signaling activity via down‐regulating the beta‐catenin and T‐cell factor 4 and lowering the level of cyclin D1 and matrix metalloproteinase 7 were observed after fisetin administration. Moreover, fisetin treatment also suppressed epidermal growth factor receptor (EGFR) and nuclear factor kappa B (NF‐kappaB) activations (Suh, Afaq, Johnson, & Mukhtar, 2009). The researchers also dose‐dependently validated the findings of anticancer role of fisetin against human colon cancer cell line HT‐29. The supplementation of fisetin at the dose of (0, 20, 40, or 60 micromol/L) significantly suppressed the both cell growth and DNA synthesis, exhibited G(2)/M phase arrest, lowered cell number, the activities of CDK‐2,4), cyclin E and D1, enhanced the p21(CIP1/WAF1) levels as well as shifting of phosphorylation state of the retinoblastoma proteins shifted from hyperphosphorylated to hypophosphorylated, decreased and an increase in. In fisetin‐treated cells, it also decreased cell division cycle protein levels (CDC)2 and CDC25C, and CDC2 activity (Lu et al., 2005).

### 2.5 Liver

Fisetin has been known to prevented from the liver cancer cell line proliferation in vivo study of mice via inhibiting the migration and invasion, inducing apoptosis, activating caspase‐3, suppressing TGF‐β1, lowering epithelial–mesenchymal transition (EMT), ameliorating cancer progression, enhancing survival time, and down‐regulating the expressions of p‐ERK1/2, vascular endothelial growth factor receptor 1(VEGFR1), p38, and pJNK, respectively (Liu, Long, Miao, Liu, & Yao, 2017). A study addressed by Chen et al. (2002) determined the cytotoxic effect of fisetin against hepatocellular carcinoma cell SK‐HEP‐1. Fisetin (80 microM) showed dose‐dependently caused DNA fragmentation, induced cellular swelling and apoptotic death, and showed characteristics of apoptosis. Moreover, fisetin also induced caspase‐3/CPP32 activity, but not of caspase‐1 activity. The cleavage of caspase‐3 substrates including D4‐GDI protein and poly(ADP‐ribose) polymerase (PARP) reduction in pro‐caspase‐3 proteins, and enhancement of p53 protein were reported after fisetin treatment in SK‐HEP‐1 cells (Chen et al., 2002). Fisetin treatment at the dose of 0.1 to 10 microM in dose‐ and time‐dependent manner in cells significantly induced quinone oxidoreductase (QR) activity associated with QR mRNA expression and activated the ARE/EpRE (Hou et al., 2001).

### 2.6 Kidney

Being a potent anticancer agent, fisetin administration in in vitro and in vivo studies in kidney renal stem cells (HuRCSCs) effectively inhibited cancer cell stages such as proliferation, cell division, and invasion as well as lowered the TET1 expression levels. It also inhibits 5hmC modification levels at the CpG islands in cyclin Y (CCNY) and CDK16 and reduces the transcription and activity (Si et al., 2019). A study has shown that fisetin treatment prevented from the proliferation of human renal carcinoma (Caki) cells through multiple processes such as (i) induction of sub‐G1 population and cleavage of poly(ADP‐ribose) polymerase (PARP), (ii) activation of caspase, (iii) caused apoptotic cell death, (iv) down‐regulation of DR5 by siRNA blocked fisetin‐induced apoptosis, (v) induction of p53 protein expression through up‐regulation of protein stability, and (vi) caused up‐regulation of (C/EBP) homologous protein (CHOP) expression and reactive oxygen species production, respectively (Min, Nam, & Kwon, 2017).

### 2.7 Bone

Fisetin in different bone cancer cell lines such as Saos‐2, MG‐63, and U2OS significantly lowered the colony formation but not in MG‐63 cells. Fisetin treatment at the rate of 40 and 20 µM showed significant reduction in cell proliferation in MG‐63 and Saos‐2 cells. It also caused G2 phase cell cycle arrest with 50 µM for 48 hr. Fisetin enhanced % cells in G2 phase and reduced % cells in G1 phase as well as reduced the stages of cyclins B1 and E1 (de Oliveira et al., 2018). In another study reported by Jang et al. (2005), they found that fisetin administration to human osteosarcoma (HOS) cells significantly inhibited growth, induced apoptosis, cleavaged PARP, activated the caspase‐8 and Bax, suppressed the Bcl‐2 levels, and released cytochrome c (Jang et al., 2005).

### 2.8 Oral

Fisetin enhances the suppression of autophagy and induces apoptotic cell death in human tongue squamous cell line Ca9‐22 (Park et al., 2019). Considering anticancer role, fisetin has been found to exhibit effectual role against different human oral squamous cell including Tca‐8113 and UM‐SCC‐23 cancer cell lines through multiple pathways, that is, suppression of Met/Src signaling pathways, inhibition of level of a disintegrin and metalloproteinase 9 (ADAM9) protein, and reduction in basal expression of Met and Src protein, respectively (Li, Qin, & Dai, 2017). In in vitro study of HSC3 human oral cancer cell lines, the administration of fisetin was associated with induction of apoptotic cell death via enhancement of reactive oxygen species and Ca^2+^, caspase‐3, caspase‐8, and caspase‐9 activities along with the reduction in mitochondrial membrane potential. The reduction in antiapoptotic protein such as BCL‐X and BCL‐2; increment of apoptotic‐associated protein expressions like BCL2‐associated X (BAX), antagonist/killer (BAK), and B‐cell lymphoma 2 (BCL2); and enhancement of apoptosis‐inducing factor (AIF), caspase‐3, caspase‐8, and caspase‐9 (cleaved forms), and cytochrome c, and endonuclease G (ENDO G) from mitochondria into the cytoplasm, respectively, were reported after fisetin treatment (Shih et al., 2017). Multiple mechanisms are involved to prevented from the human oral cancer SCC‐4 cells after administration of fisetin in in vitro study. These mechanisms include induction of cell death via morphological changes, promotion of ROS and Ca^2+^ production, reduction in expressions of ΔΨ_m_, and enhancement of caspase‐3, caspase‐8, and caspase‐9 activities, and caused G2/M phase arrest, respectively. It also caused lowering in levels of Bcl‐2 (antiapoptotic proteins) and enhancement in expressions of pro‐apoptotic proteins (Bax and Bid) as well as enhancement in the AIF, cytochrome c, and Endo G secreted from mitochondria. Furthermore, cell death in SCC‐4 cells was done through endoplasmic reticulum stress (Su et al., 2017). The role of fisetin on TU212 cell proliferation was reported by Zhang and Jia (2016), and they investigated that fisetin induced apoptosis, suppressed cell proliferation, and promoted caspase‐3 expressions by regulating PI3K/AKT/NF‐κB and inhibiting the activation of PI3K/AKT‐regulated mTOR. Moreover, fisetin administration in in vivo study markedly lowered tumor volume and weight in nude mice (Zhang & Jia, 2016). A research work conducted by Li et al. explored the chemopreventive role of fisetin bioactive compound against nasopharyngeal carcinoma (NPC) cells through the suppression of the survival rate of CNE‐latent membrane protein 1 (LMP1) cells and NF‐κB activation, preventing the nuclear translocation of NF‐κB (p65) and IκBα phosphorylation as well as suppressing the cyclin D1 expressions. Li, Liang, et al. (2014) showed that they strengthened the previous results regarding anticancer role of fisetin against nasopharyngeal carcinoma (NPC) cells including CNE1‐LMP1 cells. Fisetin treatment suppresses the migration and invasion, inhibits molecular changes linked with epithelial–mesenchymal transition (EMT) induced by LMP1, down‐regulates the mesenchymal marker and vimentin protein levels, and up‐regulates the E‐cadherin protein and epithelial marker. Further, it also lowers the levels of twist protein, an EMT regulator (Li, Park, & Kim, 2015).

### 2.9 Stomach

Fisetin bioactive compound exerts anticancer activity against SGC7901 cancer and GES‐1 normal cells by acting on cellular events. Resultantly, different doses of fisetin at the rate of 1, 5, 10, 15, and 20 µM for 48h in dose‐dependent manner significantly decreased the proliferation, enhanced the apoptosis, and reduced the activation of ERK 1/2, accordingly (Yan, Chen, Zhao, & Ye, 2018). Fisetin was also reported to prevented from the human gastric carcinoma AGS and SNU‐1 cells by applying different concentrations (25–100 μM). It markedly increases the levels of p53 and its S15 phosphorylation, enhances apoptotic cells, lowers the levels of G1 phase cyclins and CDKs, exhibits mitochondrial membrane depolarization, and enhances the expressions of p53 and its S15 phosphorylation (Sabarwal, Agarwal, & Singh, 2017).

Yan demonstrated that treatment of different doses of fisetin (1, 5, 10, 15, and 20 µM) in SGC7901 cancer and GES‐1 normal cells in a dose‐dependent fashion markedly lowered the proliferation rate, increased apoptosis, and reduced the activation of ERK ½ (Yan et al., 2018).

### 2.10 Blood

The effect of fisetin was analyzed on human Burkitt's lymphoma Raji cells through multiple processes including induction of apoptotic cell death, suppression of mammalian target of rapamycin (mTOR), and aberrant regulation of phosphatidylinositol‐3‐kinases (PI3Ks) (Lim et al., 2015). A study performed by Sak, Kasemaa, & Everaus, 2016 examined that administration of fisetin (37 μM) showed enhancement in the apoptotic cell population by enhancing the activities of caspase‐3 and caspase‐9 and triggering the intrinsic apoptotic pathway in lymphocytic leukemia (CLL) lines, EHEB and HG‐3, and increased (Sak et al., 2016). Similarly, fisetin in human K562 CML cells dose‐dependently caused inhibition of cell proliferation triggering programmed cell death. The enhancement of caspase‐3 activation and mitochondrial depolarization caused S and G2/M cell cycle arrests and G0/G1 arrest, and alteration of multiple pathways (KIT receptor signaling, JAK/STAT pathway, and growth hormone receptor signaling) was also found upon fisetin treatment. Furthermore, modulation of expressions of genes, which are participated in cell division and cell proliferation, apoptosis, cell cycle regulation, and many other cellular processes (transcription, translation, and replication), was observed after fisetin treatment (Adan & Baran, 2016). A study stated by Adan and Baran explored the anticancer role of fisetin flavonoid in a concentration‐ and time‐dependent fashion against human HL60 acute promyelocytic leukemia cells through various pathways such as inhibition of proliferation, disrupting the potential of mitochondrial membrane, induction of G2/M arrest and G0/G1 arrest, enhancement of caspase‐3 activity, and alteration in mitogen‐activated protein kinases (MAPK), and inhibitor of DNA‐binding (ID) signaling pathways, respectively (Adan & Baran, 2015). Multiple studies found that in in vivo study, fisetin killed the THP‐1 cells in the mouse xenograft model through tumor shrinkage; suppression of the downstream components of the mTORC1 pathway; induction of hypophosphorylation of eIF4B, S6 Ri P kinase, and eEF2K; down‐regulation of levels of p70 S6 kinase; enhancement of production of NO; and elevation of Ca^+2^ entry activating the caspase‐dependent apoptotic pathways (Ash, Subramanian, Surolia, & Shaha, 2015; Monasterio, Urdaci, Pinchuk, Lopez‐Moratalla, & Martinez‐Irujo, 2004). Previous study also reported the anticancer role of fisetin in dose‐dependent fashion against human leukemia cell line (HL‐60). Fisetin induced the caspase‐3/CPP32 activity, cleavaged poly(ADP‐ribose) polymerase (PARP), and lowered pro‐caspase‐3 protein. It also decreased the antiapoptotic protein (Mcl‐1) and increased the pro‐apoptotic protein (bax). Additionally, fisetin also enhanced the endonuclease activity and lowered the endogenous ROS production (Lee et al., 2002). Therefore, fisetin also induces apoptosis, enhances the fraction of the cells with sub‐G1 content, up‐regulates the Bax, Bim, and Bad, activates the caspase‐3, down‐regulates the Bcl‐2 and Mcl‐1(L), activates the adenosine monophosphate‐activated protein kinase (AMPK) along with its substrate acetyl‐CoA carboxylase (ACC), stimulates the production of ROS, and decreases the phosphorylation of AKT and mTOR, respectively.

### 2.11 Lung

In non‐small‐cell lung carcinoma (NSCLC) cells A549 and H1299, fisetin showed major restriction in the stages of migration and invasion. It also attenuated the EMT expression with up‐regulating the expression of epithelial marker E‐cadherin and concomitantly down‐regulating mesenchymal markers vimentin as well as N‐cadherin along with invasion marker MMP‐2. In addition, down‐regulation of CD44 and CD133 markers and reduction in levels of signaling proteins including EGFR, NF‐κB, STAT‐3, and β‐catenin were done after fisetin treatment (Tabussam et al.,, 2020). Fisetin in combination with paclitaxel prevents from the cell migration, proliferation, and invasion of A549 human non‐small‐cell lung cancer (NSCLC) cells (Klimaszewska‐Wiśniewska, Hałas‐Wiśniewska, Grzanka, & Grzanka, 2018). In another study conducted by Wang and their coworkers, they reported that reduction in levels of B‐cell lymphoma 2, c‐myc, cyclooxygenase‐2, metalloproteinase‐2/9, CXC chemokine receptor type 4, cyclin D1, cluster of differentiation 44, and cyclin‐dependent kinase inhibitor (CDKN) 1A/B, and enhancement in levels of CDKN2D and E‐cadherin caused increment in activity of caspase‐3/caspase‐9 via targeting the extracellular signal‐regulated kinase signaling pathway; these changes were reported after fisetin treatment in the A549 cell line. Likewise, in vivo role of fisetin against lung cancer cell lines of mouse xenograft model was associated with induction of apoptosis, promotion of caspase‐3 signaling pathway, reduction in antiapoptotic proteins (Bcl‐2 and Bcl‐xl), enhancement in pro‐apoptotic proteins (Bax and Bad), and enhancement in the death receptor (DR) of tumor necrosis factor‐related apoptosis‐inducing ligand (TRAIL), respectively (Shi, Wang, Meng, Chen, & Meng, 2017). The earlier studies conducted by Zhang and their colleagues explicated that fisetin markedly enhanced the sensitivity of erlotinib‐resistant lung cancer cells HCC827 to erlotinib via suppressing the aberrant activation of MAPK and AKT signaling pathways resulted from AXL suppression (Klimaszewska‐Wisniewska et al., 2016; Zhang et al., 2016). In this connection, fisetin (75 μg/mL) dose‐dependently showed cytotoxic effect against human NCI‐H460 cells through multiple approaches such as induction of production of intracellular reactive oxygen species and apoptosis; suppression of cell viability and mitochondrial membrane depolarization; enhancement in sub‐G1 phase cells; modulation of expression of apoptosis‐associated proteins; and reduction in expression of B‐cell lymphoma 2. In addition, it also enhanced the Bcl‐2‐associated X protein and activated the caspase‐3 and caspase‐9, respectively (Kang et al., 2016).

The research conducted by Kang and their coworkers investigated the chemopreventive role of fisetin against the proliferation of NCI‐H460 cells. The induction of mitochondrial reactive oxygen species, endoplasmic reticulum (ER) stress‐mediated apoptosis, and ER stress characteristic signs: expression of ER stress‐related proteins; ER staining; phosphorylation of protein kinase RNA (PKR)‐like endoplasmic reticulum kinase (PERK), mitochondrial Ca(2+) overload; cleavage of activating transcription factor‐6; glucose‐regulated protein (GRP)‐78, and phosphorylation of eukaryotic initiation factor‐2 (α subunit; phosphorylation of inositol‐requiring kinase‐1, and splicing of X‐box transcription factor‐1. It also cleaved caspase‐12 and C/EBP homologous protein. siRNA‐mediated knockdown of CHOP and ATF‐6 attenuated fisetin‐induced apoptotic cell death. Moreover, induction of phosphorylation of ERK, JNK, and p38 MAPK was also reported by fisetin treatment (Kang, Piao, & Hyun, 2015). Fisetin in cisplatin‐resistant A549‐CR cells inhibits the aberrant activation of MAPK signaling pathways (Ravichandran et al., 2014; Zhuo, Zhang, Zhu, Zhu, & Chen, 2015). Considering the potential and significant role of fisetin against human non‐small‐cell lung cancer (NSCLC) cells (A549) in dose‐dependent fashion, inhibition of cell proliferation, PI3K/Akt, and mTOR signaling, reduction in development of colonies, inhibition of phosphorylation (mTOR, Akt, eIF‐4E, p70S6K1, and 4E‐BP1), reduction in protein expression of PI3K (p85 and p110), and also suppression of the constituents of mTOR signaling complex (Raptor, Rictor, PRAS40, and GβL) were reported after fisetin administration. Cells treated with fisetin compound showed substantial decrease in the phosphorylation of TSC2 and increase in the phosphorylation of AMPKα, respectively (Khan et al., 2012). Moreover, fisetin in B(a)P‐induced mice markedly restored the levels of lipid peroxidation (LPO) and antioxidants (enzymic and nonenzymic), decreased the degree of histological lesions, and enhanced the proliferating cell nuclear antigen (PCNA) (Ravichandran, Suresh, Ramesh, & Siva, 2011). The inhibitory concentration value of fisetin for Lewis lung carcinoma cells (LLCs) was 59 μM. The intraperitoneal administration of fisetin at the rate of 223 mg/kg caused 66% tumor growth suppression and reduction in microvessel density (Touil, Seguin, Scherman, & Chabot, 2011). A group of researchers and investigators found that fisetin has inhibitory effect on different cancer stages (adhesion, migration, and invasion) of human lung adenocarcinoma A549 cells through multiple pathways such as down‐regulation of levels of matrix metalloproteinase‐2 (MMP‐2), urokinase‐type plasminogen activator (uPA), and suppression of phosphorylation of extracellular signal‐regulated kinases 1 and 2 (ERK1/2) at both the protein and mRNA levels. Likewise, reduction in nuclear levels of nuclear factor kappa B (NF‐kappaB), c‐Jun, and c‐Fos, and prevention from the binding abilities of NF‐kappaB and activator protein‐1 (AP‐1) were done after fisetin administration.

### 2.12 Bladder

The induction of bladder cancer in experimental animals (rat) was done via intravesical N‐methyl‐N‐nitrosourea (MNU), whereas administration of fisetin bioactive compound prevented from the bladder cancer cell proliferation through inducing apoptosis, down‐regulating the NF‐κB pathway activity, up‐regulating the p53 pathway activity, and exhibiting changes in the ratio of pro‐ and antiapoptotic proteins (Li, Qu, et al., 2014). Moreover, a group of researchers and investigators have found that fisetin showed strong anticancer role in T24 and EJ cells through preventing the propagation, blocking cell cycle progression in the G0/G1 phase, and inducing apoptosis. Additionally, significant enhancement in levels of p21 and p53 proteins, caused cell cycle arrest, increment in expressions of Bax and Bak, and reduction in levels of Bcl‐2, Bcl‐xL, cyclin (A, D1), and CDK (2, 4), respectively, were reported after fisetin treatment. Further, these changes led to triggering of the mitochondrial apoptotic pathway (Li et al., 2011).

### 2.13 Ovarian

Dose‐dependently, fisetin in a xenograft mouse model carrying SKOV3 cells significantly induced increased tumor apoptosis, proliferation suppression, and antiangiogenesis activities (Xiao et al., 2018). The previous findings reported by Meng et al. *n* in vitro and in vivo studies observed that fisetin‐treated cells (ovarian cancer cell line SKOV3) markedly decreased the tumor volume, tumor mass, and Bcl‐2 levels and increased the Bax expressions in concentration‐dependent manner in athymic rude rat model (Meng et al., 2016). The effects of fisetin bioactive ingredient in SKOV‐3/PAX cells in a dose‐dependent manner are associated with suppressing tumor growth, the cleavage of caspase‐9, caspase‐8, and caspase‐3, and PARP as well as enhanced the sub‐G1 phase and lowered AKT phosphorylation (Choi et al., 2016).

In human ovarian cancer cell line SKOV3, fisetin (1.25 mg/kg) and fisetin nanoparticle (1.25 mg/kg) considerably inhibited the ovarian cancer cell in a dose‐dependent manner with half‐maximal inhibitory concentration (IC_50_) value of 125–250 μg/mL and 62.5–125 μg/mL, respectively (Guo et al., 2018).

### 2.14 Cervical

The combination of fisetin and sorafenib synergistically induced apoptosis in HeLa cells followed by enhancement in loss of mitochondrial membrane potential, activation of caspase‐3 and caspase‐8, and increment of Bax/Bcl‐2 ratio; exhibited cleavage of PARP level; and disrupted the potential of mitochondrial membrane (Lin et al., 2016). In a dose‐dependent fashion, fisetin suppresses the expression and activity of urokinase plasminogen activator (uPA), lowers the phosphorylation of p38 MAPK, inhibits the tetradecanoylphorbol‐13‐acetate (TPA)‐induced activation of p38 MAPK and uPA, suppresses the TPA‐improved migratory and invasive capabilities, and represses the promoter activity of the uPA gene. Further, fisetin intensely disrupted the nuclear translocation of NF‐κB and its binding amount to the uPA gene promoter (Chou et al., 2013). Fisetin in HeLa tumor xenograft cells in a dose‐ and time‐dependent manner has been found to induce apoptosis and trigger the activations of caspase‐3 and caspase‐8, and the cleavages of poly(ADP‐ribose) polymerase. Furthermore, fisetin activates the phosphorylation of ERK1/2 and tumor growth (Ying et al., 2012).

### 2.15 Skin

Malignant melanoma is causative around 75% of deaths, which is associated with skin cancer. Fisetin has significant effect on production of free fatty acids, tumor incidence, IL‐1α, TNF‐α, and antioxidant enzymes (glutathione and catalase contents) in ultravoilet‐induced skin cancer rats (Moolakkadath et al., 2019). YB‐1 leads to cell proliferation and invasion through promoting epithelial‐to‐mesenchymal transition. The p90 ribosomal S6 kinase (RSK) activates YB‐1 to proliferate melanoma growth. Multiple pathways are involved such as YB‐1 dephosphorylation, suppression of mesenchymal markers, reduction in transcript levels and matrix‐metalloproteinases, inhibition of RSK activity through binding to the kinase, inhibition of YB‐1/RSK signaling independent of its effect on ERK, and reduction in MDR1 levels in melanoma cells (Sechi et al., 2018). The administration of fisetin (250 and 500 nmol) in SKH‐1 hairless mouse provides protection against solar ultraviolet B (UVB) radiation skin cancer through multiple pathways such as reduction in hyperplasia and PGE2; infiltration of inflammatory cells, receptors (EP1‐EP4), COX‐2, and MPO activity; and reduction in inflammatory cytokines (TNF‐α, IL‐1β, and IL‐6) and cell proliferation markers. It also increased the p21 and p53 proteins, inhibited phosphorylation of PI3K and AKT levels, and activated NF‐κB signaling pathway (Pal, Athar, Elmets, & Afaq, 2015). BRAF gene regulates the MAPK signaling cascade along with activating mutations in the serine in human melanoma cells. On other side, MAPK pathway also activates the NF‐κB factor. Inhibition of cell invasion, reduction in phosphorylation of MEK1/2 and ERK1/2, suppression of activation of IKK, and reduction in the activation of the NF‐κB signaling pathway were reported after fisetin treatment (5–20 µM) (Pal et al., 2014).

Treatment of A431 cells with fisetin (5–80 μm) resulted in dose‐ and time‐dependent substantial decreases in cell viability. Treatment with fisetin greatly decreased colony forming in cells A431. Treatment with fisetin greatly decreased colony forming in cells A431. The treatment of A431 cells by fisetin involved in G2/M arrest and apoptosis induction. Additionally, treatment of A431 cells with fisetin resulted in (i) increased production of pro‐apoptotic proteins (Bax, Bak, and Bad); (ii) decreased production of antiapoptotic proteins (Bcl2, Bcl‐xL, and Mcl‐1); (iii) degradation of mitochondrial potential; (iv) release of cytochrome c and Smac/DIABLO from mitochondria; (v) cleavage of poly(ADP‐ribose) polymerase (PARP) protein; and (vi) caspase activation (Pal, Sharma, Elmets, Athar, & Afaq, 2013). In A375 and 451Lu human melanoma cells, fisetin induced endoplasmic reticulum (ER) stress, up‐regulated the ER stress markers (ATF4, XBP1s, IRE1α, and GRP78), and activated the extrinsic and intrinsic apoptotic pathways. Fisetin exhibited the autophagic response concomitant with induction of apoptosis, suppressed the progression, and increased cleaved caspase‐3. In resulting, fisetin treatment leads to phosphorylation and activation of the multifunctional AMP‐activated protein kinase (AMPK) (Syed, Lall, Chamcheu, Haidar, & Mukhtar, 2014).

### 2.16 Other cancers

A study described by Chen, Wu, et al. (2015) assessed that noncytotoxic dose of fisetin bioactive compound in GBM8401 cells markedly inhibited the cell migration and invasion, and phosphorylated the ERK1/2 that suppressed the levels of ADAM9 protein and mRNA. In another study reported by Kim and their coworkers, they explored that fisetin in combination with gemcitabine has anticancer role on human cholangiocarcinoma (CCA) cell line SNU‐308. Both compounds suppress the survival rate of cells via phosphorylating ERK and induce apoptotic cell death, and also decreased the levels of cellular proliferative markers (myelocytomatosis and phospho‐p6) (Kim et al., 2016).

## 3 CONCLUSION AND FUTURE PERSPECTIVE

Many efforts have been made on researching potential anticancer agents in last decades. Natural products were among the popularly investigated agents. They are considered to have wider targeting pathways in comparison with synthetic drugs. Fisetin as a polyphenol with pleiotropic pharmacological properties showed promising anticancer activity in a wide range of cancers. Fisetin suppresses the cancer cell stages, prevents progression in cell cycle and cell growth, and induces apoptosis, Further, fisetin suppresses the activation of the PKCα/ROS/ERK1/2 and p38 MAPK signaling pathways, reduces the NF‐κB activation, and down‐regulates the level of the oncoprotein securin. Specific research work is required to further explore its toxicity and interaction with current chemotherapeutic agents as well as potential efficacy in clinical setting.

4

**TABLE 1:** Anticancer perspectives of fisetin

Cancer types	Mechanisms	Cell lines	References
Breast	Induced the proteasomal degradation Decreased enolase phosphorylation Altered the phosphatidylinositol 3‐kinase/Akt signaling	MCF‐7	Guo et al. (2018)
	Suppressed the cell proliferation, migration, and invasion Inhibited phosphoinositide 3‐kinase (PI3K)‐Akt‐GSK‐3β signaling pathway Up‐regulated expression of PTEN mRNA and protein	MDA‐MB‐231 and BT549 cells	Li et al. (2018)
	Inhibited the different cancer stages (migration and invasion) Suppressed the metastasis and invasiveness, induction of the apoptosis Regulation of the phosphatidylinositol‐3‐kinase/protein kinase B/mammalian target of rapamycin pathway	MDA‐MB‐231, MCF‐7, and 4T1	Sun et al. (2018)
	Increased HO‐1 mRNA, protein expressions, elevated Nrf2 expression, abrogated the HO‐1 expression Decreased MMP‐2 and MMP‐9 enzyme activity and gene	4T1 and JC	Tsai et al. (2018)
	Decreased the development and induced apoptotic cell death	MCF‐7	Pawar et al., 2018)
	Suppressed tumor burden, migration, and volume	MCF‐7	Wang et al., 2017)
Prostate	Decreased cell viability and metastatic properties Inhibited the cancer stages (proliferation, tumor growth, and invasion)	PC‐3M‐luc‐6, 22Rν1, and C4‐2	Mukhtar et al. (2016)
	Down‐regulation of intracellular and secreted hyaluronan levels Suppressed the hyaluronan (HA) synthesis and degradation enzymes	PCa	Lall et al. (2016)
	Inhibition of viability and colony formation Inhibition of protein Nudc	PCa?	Mukhtar et al. (2015)
	Inhibited EGF‐induced YB‐1 phosphorylation and EMT markers	PCa	Khan et al. (2014)
Pancreatic	Inhibited cell proliferation and enhanced AMPK/mTOR signaling pathway Increased stress‐induced transcription factor p8, ATF4, and ATF6	PANC‐1	Jia et al. (2019)
	Induced apoptotic cell death and activated caspase‐3/caspase‐7 Reduced ERK‐induced MYC instability at the protein level, down‐regulated ERK at the protein and mRNA levels Suppressed extracellular signal‐regulated kinase (ERK)‐v‐myc avian myelocytomatosis viral oncogene homolog (MYC) pathway	MIA PaCa‐2	Kim et al. (2018; Youns and Abdel Halim Hegazv, (2017)
Colon	Suppressed cell viability and tumor growth, and induced apoptosis Promoted caspase‐8 and cytochrome c expressions Suppressed the aberrant activation of IGF1R and AKT proteins	LoVo cells	Jeng et al. (2018)
	Down‐regulated the level of the oncoprotein securin	CT‐26, HCT116	Leu et al. (2016)
Liver	Inhibited the migration and invasion and induced apoptosis Suppressed TGF‐β1 and lowered epithelial–mesenchymal transition Down‐regulated the expressions of p‐ERK1/2, VEGFR1, p38, and pJNK	HCC‐LM3 and HepG2	Liu et al. (2017)
Renal	Lowered the TET1 expression levels and inhibited 5hmC modification levels	HuRCSCs	Si et al. (2019)
Bone	Caused G2 phase cell cycle arrest and reduced the levels of cyclins B1 and E1	Saos‐2, MG‐63, and U2OS	de Oliveira et al. (2018)
Oral	Enhanced suppression of autophagy and induced apoptotic cell death	Ca9‐22	Park et al. (2019)
	Suppressed Met/Src signaling pathways Inhibited disintegrin and metalloproteinase 9 (ADAM9) protein level Lowered basal expression of Met and Src protein	Tca‐8113 and UM‐SCC‐23	Li et al. (2017)
Stomach	Enhanced the apoptosis and reduced the activation of ERK 1/2	SGC7901, GES‐1	Yan et al. (2018)
	Increased the levels of p53 and its S15 phosphorylation Lowered the levels of G1 phase cyclins and CDKs Enhanced expressions of p53 and its S15 phosphorylation	AGS, SNU‐1	Sabarwal et al. (2017)
Blood	Increased the activities of caspase‐3 and caspase‐9 Triggered intrinsic apoptotic pathway	EHEB, HG‐3	Sak et al. (2016)
	Caused S and G2/M cell cycle arrests and G0/G1 arrest Altered KIT receptor signaling, JAK/STAT pathway, and growth hormone receptor signaling	K562 CML cells	Adan et al. (2016)
	Suppressed mammalian target of rapamycin (mTOR) and aberrant regulation of phosphatidylinositol‐3‐kinases (PI3Ks)	Burkitt's lymphoma Raji	Lim et al. (2015)
Lung	Attenuated the EMT expression with up‐regulating the expression of epithelial marker E‐cadherin Down‐regulated the mesenchymal markers vimentin as well as N‐cadherin along with invasion marker MMP‐2 Down‐regulated the markers (CD44 and CD133) and NF‐κB, EGFR, β‐catenin, STAT‐3	A549, H1299	Tabasum and Singh (2019)
	Prevented from the proliferation, cell migration, and invasion	A549	Klimaszewska‐Wiśniewska et al. (2018)
	Lowered B‐cell lymphoma 2, c‐myc, cyclooxygenase‐2, metalloproteinase‐2/9, CXC chemokine receptor type 4, cyclin D1, and cluster of differentiation 44	A549	Wang & Huang, 2018)
Ovarian	Induced increased tumor apoptosis and proliferation suppression	SKOV3	Xiao et al. (2018)
	Decreased the tumor volume, tumor mass, and Bcl‐2 levels and increased the Bax expressions	SKOV3	Meng et al. (2016)
Cervical	Induced apoptosis and exhibited cleavage of PARP level Disrupted mitochondrial membrane potential	HeLa	Lin et al. (2016)
